# Reconstruction of a Nearly Complete *Pseudomonas* Draft Genome Sequence from a Coalbed Methane-Produced Water Metagenome

**DOI:** 10.1128/genomeA.01024-16

**Published:** 2016-10-06

**Authors:** Daniel E. Ross, Djuna Gulliver

**Affiliations:** a Office of Research & Development, National Energy Technology Laboratory, Pittsburgh, Pennsylvania, USA; b AECOM, Pittsburgh, Pennsylvania, USA

## Abstract

The draft genome sequence of *Pseudomonas stutzeri* strain K35 was separated from a metagenome derived from a produced water microbial community of a coalbed methane well. The genome encodes a complete nitrogen fixation pathway and the upper and lower naphthalene degradation pathways.

## GENOME ANNOUNCEMENT

The *Pseudomonas stutzeri* strain K35 draft genome was extracted *in silico* from the metagenome of a produced water sample, which was taken from the Pocahontas 3 coal seam in the Central Appalachian Basin at a depth of 1,912 ft. Genomic DNA was isolated using the PowerSoil DNA kit (MoBio Laboratories, Inc., Carlsbad, CA). A paired-end (2 × 250) library was prepared using the Nextera XT kit and the MiSeq reagent kit v2 (Illumina, Inc., San Diego, CA) and run with 500 cycles. The resultant 430,878 reads were assembled with metaSPAdes (version 3.7.0) (1). The metagenome assembly yielded 12,264 total contigs (12,332,388 bp), with an *N*_50_ of 4,275 and a GC% of 56.52 (SRA accession no. SRP079849). The metagenome was binned with VizBin (2) and 22.5% of the contigs were assigned to the genus *Pseudomonas*. The metagenome reads were mapped to the contigs of the *Pseudomonas* genome bin using CLC genomics workbench (Qiagen) and mapped reads were reassembled with SPAdes (default parameters; contigs added as trusted contigs). The final contigs were extended using paired-read iterative contig extension (PRICE) (3).

The genome was 99.2% complete, with 0.75% contamination, and 0% strain heterogeneity using 833 marker genes specific to the genus *Pseudomonas* in CheckM (4). The longest contig was 190,857 bp with an *N*_50_ of 47,412 and GC% of 62.94. The *Pseudomonas* genome bin contained a total of 194 contigs (4,800,235 bp) and mapped best to *Pseudomonas stutzeri* strain AN10 (CCUG 29243) (accession no. NC_018028) (5) with 167 contigs (4,570,745 bp) mapping at an e-value cutoff of 1e-100 (6). The average nucleotide identity (ANI) and average amino acid identity (AAI) were determined for *P. stutzeri* strain K35 and 30 publically available genomes (complete and draft). ANI and AAI values were highest for *P. stutzeri* strain BAL361 (98.11% and 98.36%), followed by *P. stutzeri* strain AN10 (CCUG 29243) (96.75% and 96.62%) and *P. stutzeri* strain ST-9 (96.56% and 96.70%).

The draft genome bin contained seven contigs (139,857 bp) that mapped to other *Pseudomonas* plasmids (BLASTn best hit) and may be part of a genomic island (GI) within the chromosome or an extrachromosomal plasmid. The GI/plasmid encodes for numerous type IV secretion system proteins, DNA replication machinery (e.g., DNA helicase, DNA topoisomerase), and mobilization elements (e.g., transposases and integrases).

The genome was annotated with Rapid Annotations using Subsystem Technology (RAST) version 2.0, using the RASTtk pipeline (7, 8), and the NCBI Prokaryotic Genomes Automatic Annotation Pipeline (http://www.ncbi.nlm.nih.gov/genome/annotation_prok/). Initial analysis revealed the presence of complete nitrogen fixation and denitrification pathways. The upper and lower naphthalene degradation pathways were also found. The genome encodes for alginate biosynthesis pathways and a number of genes involved in oxygen stress (catalases, peroxidases, and superoxide dismutases). This genome provides a platform for understanding the role *Pseudomonas* spp. play in the conversion of coal to methane and the ecology of coalbed methane systems.

### Accession number(s).

This whole-genome shotgun project has been deposited at DDBJ/ENA/GenBank under the accession no. MCAL00000000. The version described in this paper is version MCAL01000000.
